# Supplementary material for: Dehydration and insulinopenia are necessary and sufficient for euglycemic ketoacidosis in SGLT2 inhibitor-treated rats
Source: Nat Commun. 2019 Feb 1;10:548. doi: 10.1038/s41467-019-08466-w (PMC6358621; doi:10.1038/s41467-019-08466-w)
Supplement: Supplementary file 1 — Supplmentary Information [file 41467_2019_8466_MOESM1_ESM.docx]

**Supplementary Figure 1.** Dapagliflozin causes ketoacidosis due to volume depletion. (A)-(B) Urine glucose and β-OHB concentrations. (C) β-OHB clearance. (D) Plasma NEFA concentrations. (E) Whole-body palmitate turnover. (F) Whole-body glycerol turnover. (G) Liver malonyl-CoA concentrations. (H) Endogenous glucose turnover. In all panels, data are the mean±S.E.M., with comparisons by ANOVA with Bonferroni’s multiple comparisons test.

**Supplementary Figure 2.** Dapagliflozin-induced ketoacidosis is associated with increased catecholamine and corticosterone concentrations due to volume depletion. (A)-(F) Plasma angiotensin II, norepinephrine, ACTH, leptin, glucagon, and growth hormone concentrations. Data are the mean±S.E.M., with comparisons by ANOVA with Bonferroni’s multiple comparisons test.

**Supplementary Figure 3.** Furosemide causes dehydration but does not lower plasma glucose/insulin or cause ketosis in recently fed (i.e. 8 hr fasted) rats. (A)-(G) Plasma angiotensin II, ADH, norepinephrine, corticosterone, insulin, glucagon, and growth hormone concentrations. (H)-(I) Plasma and urine β-OHB concentrations. (J) β-OHB clearance, (K) Plasma NEFA. (L) Whole-body fatty acid turnover. (M) Whole-body glycerol turnover. (N)-(O) Liver acetyl- and malonyl-CoA concentrations. (P) Whole-body glucose turnover. In all panels, data are the mean±S.E.M., with comparisons by the 2-tailed unpaired Student’s t-test.

**Supplementary Figure 4.** Infusion of glucose to increase plasma glucose concentrations in dapagliflozin-treated rats to concentrations measured in controls suppresses ketogenesis. (A)-(C) Plasma glucose, insulin, and β-OHB concentrations. (D)-(F) Whole-body β-OHB, fatty acid, and glycerol turnover. (G) Endogenous glucose turnover. (H)-(K) Plasma epinephrine, norepinephrine, corticosterone, and glucagon concentrations. In all panels, data are the mean±S.E.M. of n=5 per group, with comparisons by the 2-tailed paired Student’s t-test.

**Supplementary Figure 5.** Dehydration causes ketoacidosis in insulinopenic rats. (A) Weight change 6 hr after treatment with furosemide in 48 hr fasted rats not given access to drinking water. (B) Heart rate. (C) Rectal temperature. (D)-(G) Plasma corticosterone, epinephrine, norepinephrine, and NEFA concentrations. (H)-(I) Whole-body fatty acid and glycerol turnover. (J) Liver acetyl-CoA concentration. (K)-(L) Plasma glucose and whole-body glucose turnover. (M)-(O) Plasma insulin, glucagon, and β-OHB concentrations. (P) Whole-body β-OHB turnover. (Q) Plasma bicarbonate. In all panels, groups were compared using the 2-tailed unpaired Student’s t-test.

**Supplementary Figure 6.** Dapagliflozin causes hyperglucagonemia but not ketoacidosis through a central mechanism. (A)-(B) Insulin and glucagon secretion in isolated rat islets incubated in dapagliflozin or canagliflozin. (C)-(H) Insulin and glucagon secretion in isolated human islets incubated in dapagliflozin or canagliflozin, cultured in either KRB or DMEM. **P*<0.05 vs. DMSO; §*P*<0.05, §§*P*<0.01 vs. dapagliflozin. In panels (A)-(H), data are the mean±S.E.M. of n=4 replicates per condition, with data compared by ANOVA with Bonferroni’s multiple comparisons test.

**Supplementary Figure 7.** Dapagliflozin causes hyperglucagonemia but not ketoacidosis through a central mechanism. (A)-(B) Weight change and urine glucose concentrations 2 hrs after an ICV injection of dapagliflozin. (C)-(G) Plasma glucagon, epinephrine, norepinephrine, glucose, and insulin concentrations. (H)-(J) Whole-body glucose, fatty acid, and glycerol turnover. (K)-(L) Plasma β-OHB concentrations and whole-body β-OHB turnover. (M) Plasma bicarbonate concentrations. Data are the mean±S.E.M., with comparisons by the 2-tailed unpaired Student’s t-test.

**Supplementary Figure 8.** Dapagliflozin causes hyperglucagonemia but not ketoacidosis through a central mechanism. (A) Weight change in rats injected with dapagliflozin 1 week following either sham surgery or unilateral vagotomy. (B)-(D) Plasma glucagon, β-OHB, and bicarbonate concentrations. Data are the mean ± S.E.M.

**Supplementary Figure 9.** Dapagliflozin causes ketoacidosis in a rat model of type 2 diabetes. (A) Urine glucose concentrations. (B)-(G) Plasma angiotensin II, ADH, insulin, glucagon, norepinephrine, and corticosterone concentrations. (H) Mean arterial pressure. (I) Plasma NEFA concentrations. (J) Whole-body glycerol turnover. (K) Plasma β-OHB concentrations. (L) Whole-body glucose turnover. Data are the mean ± S.E.M., with comparisons by ANOVA with Bonferroni’s multiple comparisons test.

**Supplementary Figure 10.** Dapagliflozin causes ketoacidosis in normal rats through a combination of β-1 adrenergic and glucocorticoid activity. (A) Urine glucose concentrations. (B) Weight change. (C) Mean arterial pressure. (D)-(F) Plasma glucose, NEFA, insulin, glucagon, epinephrine, norepinephrine, and corticosterone concentrations. Data are the mean ± S.E.M., with comparisons by ANOVA with Bonferroni’s multiple comparisons test.
